# Presenting Influenza A M2e Antigen on Recombinant Spores of *Bacillus subtilis*

**DOI:** 10.1371/journal.pone.0167225

**Published:** 2016-11-30

**Authors:** Tomasz Łęga, Paulina Weiher, Michał Obuchowski, Dawid Nidzworski

**Affiliations:** 1 Department of Medical Biotechnology, Intercollegiate Faculty of Biotechnology, University of Gdańsk and Medical University of Gdańsk, Gdańsk, Poland; 2 Department of Recombinant Vaccine, Intercollegiate Faculty of Biotechnology, University of Gdańsk and Medical University of Gdańsk, Gdańsk, Poland; 3 Department of Medical Biotechnology, Intercollegiate Faculty of Biotechnology UG-GUMed, Medical University of Gdańsk, Gdańsk, Poland; 4 Department of Recombinant Vaccine, Intercollegiate Faculty of Biotechnology, University of Gdańsk and Medical University of Gdańsk, Gdańsk, Poland; Monash University, Australia, AUSTRALIA

## Abstract

Effective vaccination against influenza virus infection is a serious problem mainly due to antigenic variability of the virus. Among many of investigated antigens, the extracellular domain of the M2 protein (M2e) features high homology in all strains of influenza A viruses and antibodies against M2e and is protective in animal models; this makes it a potential candidate for generation of a universal influenza vaccine. However, due to the low immunogenicity of the M2e, formulation of a vaccine based on this antigen requires some modification to induce effective immune responses. In this work we evaluated the possible use of *Bacillus subtilis* spores as a carrier of the Influenza A M2e antigen in mucosal vaccination. A tandem repeat of 4 consensus sequences coding for human—avian—swine—human M2e (M2eH-A-S-H) peptide was fused to spore coat proteins and stably exposed on the spore surface, as demonstrated by the immunostaining of intact, recombinant spores. Oral immunization of mice with recombinant endospores carrying M2eH-A-S-H elicited specific antibody production without the addition of adjuvants. *Bacillus subtilis* endospores can serve as influenza antigen carriers. Recombinant spores constructed in this work showed low immunogenicity although were able to induce antibody production. The System of influenza antigen administration presented in this work is attractive mainly due to the omitting time-consuming and cost-intensive immunogen production and purification. Therefore modification should be made to increase the immunogenicity of the presented system.

## Introduction

Influenza virus belongs to the *Orthomyxoviridae* family and because of its high virulence and adaptive abilities it is one of the world's most dangerous pathogens of warm-blooded vertebrates. There are three types of the virus: A, B, and C, though only type A viruses constitute a serious problem by causing severe symptoms during infection. It is also the type A that is responsible for epidemics and pandemics. Only in the 20th century, type A viruses caused 3 pandemics, which caused millions of deaths [1].

Most of existing vaccines owe their effectiveness to the ability to induce the production of neutralizing antibodies directed against hemagglutinin (HA) [2]. However, antibodies to HA provide potent but strain-specific virus protection. Due to the frequent antigenic drifts and antigenic shifts of the circulating virus there is a need for annual vaccine re-formulation and vaccination. This is a serious problem for influenza vaccination, which could be solved by generation of a universal vaccine able to confer cross protection against different influenza variants and subtypes. Recently studied antigens that could induce cross-reactive immune response are the conserved stalk domain of the hemagglutinin (HA2) [3,4], influenza A nucleoprotein (NP) [5,6] and the matrix 2 (M2) protein [7].

Influenza A M2 protein is very abundant in the plasma membrane of influenza infected cells and is further incorporated in smaller amounts into budding virions [8]. It has been demonstrated that the extracellular domain of the M2 (M2e) protein is highly conserved among all strains of influenza A virus. It was shown that immunization with M2e induces protection against influenza A infection in mice [9,10]. While natural influenza virus infection and available vaccines do not induce a strong M2e-specific antibody response, fusing M2e to a suitable carrier significantly increases its immunogenicity. The recently studied carrier molecules include: *Brucella abortus* lumazine synthase protein (BLS) [11], tuftsin [12], chicken C3d [13] and others [14–16].

In last years a new live antigen carrier system emerged [17]. Bacterium *Bacillus subtilis* is a gram-positive bacillus which produce endospores and is generally regarded as safe (GRAS). Spores of *Bacillus subtilis* are referred as being one of the most resistant life forms. Being metabolically dormant, spores are resistant to many environmental stressors such as UV radiation, desiccation, heat or freezing [18]. Free, mature spores are enclosed in a thick protein envelope called coat, which is made of at least 70 different protein species [19]. Spore coat is further divided into inner coat, outer coat and outermost crust. Recent studies show that spores can serve as an antigen carrier by fusing peptide of interest to spore coat proteins [20]. There is evidence that oral or intranasal administration of spores presenting antigens induces a specific, both cellular and humoral immune response, which can protect animals from infection [21,22].

**Fig 1:**
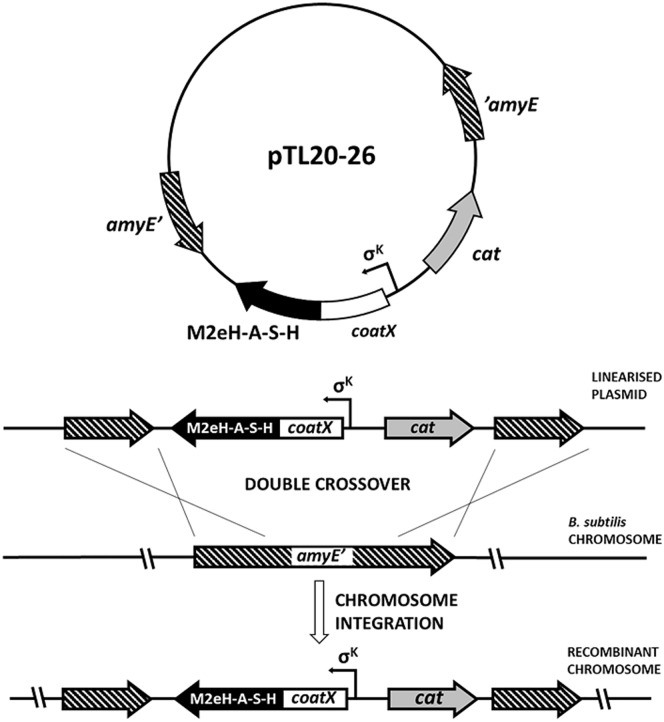
Schematic steps of building strains carrying fusion genes.

**Fig 2:**
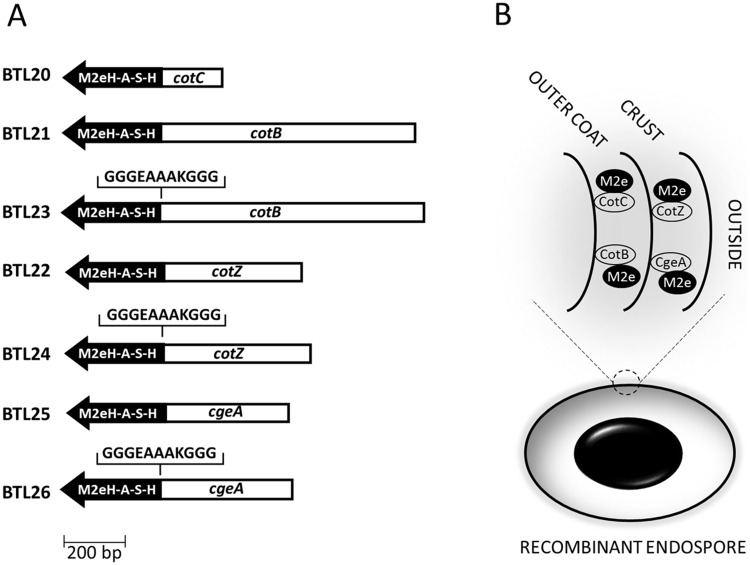
Overall idea of presenting M2e antigen on spore surface. Schematic representation of the constructed fusion genes (A). The fusion proteins, composed by a carrier (CotC, CotZ, CotB and CgeA) and a passenger peptide (M2eH-A-S-H) are exposed on the spore surface (B).

In our study we constructed *Bacillus subtilis* strains producing spores presenting M2eH-A-S-H antigen on their surface using a genetic approach (Figs 1 and 2). Recombinant spores were orally administrated to mice to evaluate the immunogenic properties of constructs. This work indicates that spores can serve as an influenza antigen carrier and induce specific antibody production without use of adjuvants.

## Materials and Methods

### Ethics statement

Efforts were made at all stages of the experiments to minimize the suffering of animals. This study was carried out in strict accordance with the recommendations in the institutional and national guidelines for animal care and use. The protocol was approved by the Committee on the Ethics of Animal Experiments of the Medical University of Gdańsk (Permit Number: 3/2014).

### Mice

BALB/c mice were purchased from the breeding facilities at the Medical University of Gdańsk. The animals were kept in polycarbonate cage, housed in well aerated rooms with a 12-h light/12-h dark cycle at 25 ± 2°C, fed with normal pellet diet and water *ad libitum*. The physical condition of the animals was monitored daily. Non of the animals exhibited clinical signs indicative of severe illness during experiment and 100% of mice reached humane endpoint euthanasia which was performed by carbon dioxide inhalation followed by cervical dislocation.

### Bacterial strains and transformation

Plasmid amplifications for nucleotide sequencing and sub-cloning experiments were performed with *Escherichia coli* DH5α [23] or BL21 strains (New England Biolabs, USA). Bacterial strains were transformed using previously described procedures: CaCl_2_-mediated transformation of *E*. *coli* competent cells [24] and transformation of *B*. *subtilis* [25]. *Bacillus subtilis* strains used in this study are listed in Table 1.

**Table 1:** Strain list.

Strain	Relevant genotype	Source or reference
***Bacillus subtilis***		
168	*trpC2*	[26]
BTL20	*amyE*::*cotC-M2eH-A-S-H*	This work
BTL21	*amyE*::*cotB-M2eH-A-S-H*	This work
BTL22	*amyE*::*cotZ-M2eH-A-S-H*	This work
BTL23	*amyE*::*cotB-gggeaaakggg-M2eH-A-S-H*	This work
BTL24	*amyE*::*cotZ-gggeaaakggg-M2eH-A-S-H*	This work
BTL25	*amyE*::*cgeA-M2eH-A-S-H*	This work
BTL26	*amyE*::*cgeA-gggeaaakggg-M2eH-A-S-H*	This work
***Escherichia coli***		
BL21(DE3)	*fhuA2 [lon] ompT gal (λ DE3) [dcm] ΔhsdS λ DE3 = λ sBamHIo ΔEcoRI-B int*::*(lacI*::*PlacUV5*::*T7 gene1) i21 Δnin5*	NEB Inc., USA
DH5α	*fhuA2 lac(del)U169 phoA glnV44 Φ80' lacZ(del)M15 gyrA96 recA1 relA1 endA1 thi-1 hsdR17*	[27]

### Construction of fusion genes

To obtain various genetic fusions genes coding for the selected coat protein were PCR-amplified together with their promoters using the *B*. *subtilis* chromosome as a template and oligonucleotide pairs cotB-F/cotB-R, cotC F/cotC-R, cgeA-F/cgeA-R and cotZ-F/cotZ-R (Table 2) as a primers for spore coat genes *cotB*, *cotC*, *cgeA* and *cotZ*, respectively. The C terminus of CotB is formed of three 27 amino acid repeats that confer genetic instability to chimeric proteins containing them [28]. For this reason, in case of CotB fusions a fragment of DNA coding for these three repeats was omitted, leaving only part of this gene encoding the N-terminal 275 amino acid residues. Amplification products were sequentially digested with *EcoR*I and *BamH*I and cloned into the pDL vector [29] obtained from Bacillus Genetic Stock Center yielding plasmids pDL-CotB, pDL-CotC, pDL-CgeA and pDL-CotZ, respectively. In the case of *cotZ*, *cotB* and *cgeA* an additional sequence coding for fusion linker GGGEAAAKGGG was added to the 3’ end of the gene using the primers pairs cotZlinker-F/cotZlinker-R, cotBlinker-F/cotBlinker-R, cgeAlinker-F/cgeAlinker-R and yielding plasmids pDL-CotZ-LINKER, pDL-CotB-LINKER, pDL-CgeA-LINKER, respectively.

**Table 2:** List of PCR oligonucleotides.

Name	Sequence	Restriction site
cotB-F	GCGGATCCGGATGATTGAT	*BamHI*
cotB-R	GATGAATTCACGGATTAGGCC	*EcoRI*
cotBlinker-F	CGCGGATCCTCCTCCACCTTTCGCTGCTGCTTCTCCTCCACCGGATGATTGATCATCTGAAG	*BamHI*
cotBlinker-R	GCCTGTTAGGAATTCCGCTCCAATCTCTTTTTACAATAGAATATATGGAACCGAAAATCATGGCGATGTATGAACGGATTAGGCC	*EcoRI*
cotC-F	GGGGATCCGTAGTGTTTTTTATGC	*BamHI*
cotC-R	CAGAATTCTGTAGGATAAATCGTTTGG	*EcoRI*
cotZ-F	GCTTAGGATCCATGATGATGTGTACGATTG	*BamHI*
cotZ-R	CGTAGCGAATTCAGTTATCACTCTTGTCCTC	*EcoRI*
cotZlinker-F	CGCGGATCCTCCTCCACCTTTCGCTGCTGCTTCTCCTCCACCATGATGTGTACGATTGAT	*BamHI*
cotZlinker-R	CCGGAATTCGCAACCCTTATTTCTACAGCAACAAATACACTCGTAGCCATCCTAGTTATCACT	*EcoRI*
cgeA-F	CGGGGATCCTGAAAAGAACGTAAC	*BamHI*
cgeA-R	CAGCTTAGAATTCTTGAGAGTGAAACATGAG	*EcoRI*
cgeAlinker-F	CGCGGATCCTCCTCCACCTTTCGCTGCTGCTTCTCCTCCACCTGAAAAGAACGTAACGCTTTC	*BamHI*
cgeAlinker-R	CCGGAATTCAAGCAGAGCCTCTGTCATCATTTAAAAAGCACCCCAGCTTACAACACTTGA	*EcoRI*
AmyA	CGAGAAGCTATCACCGCCCAGC	-
AmyS	CCAATGAGGTTAAGAGTATTCC	-

DNA sequence coding for M2eH-A-S-H peptide (MSLLTEVETPIRNEWGCRCNDSSDMSLLTEVETPTRNGWECKCSDSSDMSLLTEVETPIRNGWECRCNDSSDMSLLTEVETPIRNEWGCRCNDSSD) was synthesized (Life Technologies) with codon optimization for *Bacillus subtilis* and restriction site *BamH*I was added at the 5’ and *Sac*I, *EcoR*I at the 3’ end of the ORF. Synthesized fragment was sequentially digested with *BamH*I and *Sac*I and cloned in frame at the 3’ end of the *cotC*, *cotB*, *cotB*-LINKER, *cotZ*, *cotZ*-LNKER, *cgeA* and *cgeA*-LINKER genes carried by plasmids pDL-CotC, pDL-CotB, pDL-CotB-LINKER, pDL-CotZ, pDL-CotZ-LINKER, pDL-CgeA, pDL-CgeA-LINKER yielding plasmids pTL20, pTL21, pTL23, pTL22, pTL24, pTL25, pTL26, respectively.

### Chromosomal integration

Appropriate plasmids were linearized by digestion with a *BsmB*I restriction enzyme. Linearized DNA was used to transform competent cells of the *B*. *subtilis* strain 168. Chloramphenicol-resistant (Cm^R^) clones were the result of a double-crossover recombination event, resulting in the interruption of the non-essential *amyE* gene on the *B*. *subtilis* chromosome. All Cm^R^ clones were tested by PCR using chromosomal DNA as a template and oligonucleotides AmyS (5′-CCAATGAGGTTAAGAGTATTCC-3′ annealing +569/+590 of *amyE*) and AmyA (5′-CGAGAAGCTATCACCGCCCAGC-3′ annealing +2128/+2150 of *amyE*) to prime DNA amplification. Selected clones were called BTL20, BTL21, BTL22, BTL23, BTL24, BTL25, BTL26 and kept for further studies (Table 1).

### Preparation of spores

Sporulation was induced by the exhaustion method in Difco sporulation medium (DSM) as described elsewhere [30]. Sporulating cultures were harvested 48 h after the initiation of sporulation and purified using a lysozyme treatment to break up any residual sporangial cells, followed by washing steps in 1 M NaCl, 1 M KCl and water (twice each), as described elsewhere [31]. PMSF (0.05 M) was included to inhibit proteolysis. After the final suspension in water, spores were treated at 65°C for 1 h to destroy any residual cells. The spore suspension was titrated immediately for determination of c.f.u./mL before freezing at -20°C. We could reliably produce 7×10^10^ spores per litre of DSM culture using this method.

### Extraction of spore coat proteins

Spore coat proteins were extracted from 50 μL of spores suspensions at high density (1 × 10^10^ spores per mL) using a decoating extraction buffer as described elsewhere [32]. Measurement of extracted proteins concentration was performed using Pierce BCA Protein Assay methods (Pierce, USA).

### Western blotting analyses

Extracted spore coat proteins were separated in NuPAGE^®^ Novex^®^ 4–12% Bis-Tris pre-cast polyacrylamide gels (Life Technologies), electrotransferred on a nitrocellulose membrane using iBlot^®^ 2 Dry Blotting System (Life Technologies). Membranes where incubated with mouse monoclonal Influenza A m2 (14C2; Santa Cruz Biotechnology, cat.# sc-32238) primary antibody followed by polyclonal goat anti-mouse IgG-alkaline phosphatase (Sigma Aldrich, cat. # A3562) secondary antibodies. Western blots were visualized developing with BCIP/NBT according to the manufacturer’s instructions (Thermo Scientific).

### Antigen expression efficiency

Spore coat proteins were extracted from 50 μL of a suspensions of 5x10^8^ spores using extraction buffer as described elsewhere [32]. Measurement of extracted proteins concentrations was performed using Pierce BCA Protein Assay methods (Pierce, USA). Amounts of protein used are given in Table 3. Extracted proteins were transferred on nitrocellulose filter (Roti-NC; ROTH) using Bio-Dot^®^ apparatus (Bio-Rad). Membranes where incubated with mouse monoclonal Influenza A m2 (14C2; Santa Cruz Biotechnology, cat.# sc-32238) primary antibody followed by polyclonal goat anti-mouse IgG-alkaline phosphatase (Sigma Aldrich, cat. # A3562) secondary antibodies. Blots were visualized developing with BCIP/NBT according to the manufacturer’s instructions (Thermo Scientific). Developed blots were scanned and images were analyzed with ImageJ freeware using Dot Blot Analyzer tool.

**Table 3:** Densitometric analysis of protein expression.

Antigen source	Amount of protein/protein extract used	Relative Optical density*	Amount of M2eH-A-S-H [ng] in extracts	% of M2eH-A-S-H in total protein extracted
Purified M2eH-A-S-H	0.36 ng	490 (±7.56)	NA	NA
	0.78 ng	1020 (±10.32)	NA	NA
	1.56 ng	2017 (±8.41)	NA	NA
	3.12 ng	4320 (±4.56)	NA	NA
	6.24 ng	8760 (±12.06)	NA	NA
BTL20	0.31 μg	3735 (±5.52)	2.70	0.87
	0.62 μg	7371 (±9.45)	5.27	0.85
	1.25 μg	15468 (±3.37)	11.00	0.88
BTL21	0.31 μg	361 (±4.39)	0.31	0.10
	0.62 μg	975 (±11.98)	0.74	0.12
	1.25 μg	1866 (±11.34)	1.38	0.11
BTL23	0.31 μg	493 (±14.05)	0.40	0.13
	0.62 μg	1413(±8.44)	1.05	0.17
	1.25 μg	2573(±7.51)	1.88	0.15
BTL22	0.31 μg	2946(±5.41)	2.14	0.69
	0.62 μg	6319(±3.58)	4.53	0.73
	1.25 μg	12289(±3.35)	8.75	0.70
BTL24	0.31 μg	2771 (±7.18)	2.02	0.65
	0.62 μg	5180 (±8.47)	3.72	0.60
	1.25 μg	10876 (±6.79)	7.75	0.62
BTL25	0.31 μg	799 (±5.45)	0.62	0.20
	0.62 μg	2026 (±6.02)	1.49	0.24
	1.25 μg	4693 (±5.89)	3.38	0.27
BTL26	0.31 μg	405 (±8.41)	0.34	0.11
	0.62 μg	1238 (±7.07)	0.93	0.15
	1.25 μg	2573 (±6.76)	1.88	0.15

*Data are given as arithmetic mean with ±SD from three independent experiments

### Immunofluorescence microscopy

Samples were fixed directly in the medium as described elsewhere [33], with the following modifications: spores were suspended in TE buffer [20 mM Tris/HCl (pH 7.5), 10 mM EDTA] containing lysozyme (2 mg/mL). After 3 min of incubation, three washes in PBS (pH 7.4) were performed before blocking with 3% BSA (w/v) in PBS for 30 min at room temperature and washing another three times in PBS. Samples were incubated overnight at 4°C with mouse monoclonal Influenza A m2 (14C2; Santa Cruz Biotechnology, cat.# sc-32238) primary antibodies, washed three times and then incubated with anti-mouse Cy3 (Jackson Immuno Research) overnight at 4°C. After three washes with PBS, samples were loaded on microscope slides previously coated with poly-L-lysine (Sigma). The coverslips were mounted on a microscope slide and viewed using a Zeiss Axioplan fluorescence microscope with the same exposure time for all samples.

### Displayed antigen accessibility

10^10^ spores were suspended in 2 mL TE buffer [20 mM Tris/HCl (pH 7.5), 10 mM EDTA] containing lysozyme (2 mg/mL). After 3 min of incubation at room temperature, three washes in PBS (pH 7.4) were performed before blocking with 3% BSA (w/v) in PBS for 30 min at room temperature and washing another three times in PBS. Samples were incubated overnight at 4°C in 2 mL PBS with mouse monoclonal Influenza A m2 (14C2; Santa Cruz Biotechnology, cat.# sc-32238) primary antibodies diluted 1:100, washed five times with 2 mL PBS and then incubated 2 h at room temperature with goat anti-mouse IgG peroxidase conjugate (Sigma Aldrich, product # A 4416) diluted 1:10 000 in 2 mL PBS. Samples were washed five times with 2 mL PBS and adjusted to 10^7^ spores/mL. 1 μL of spore suspension was added to the 100 μL of 3,3’,5,5’–Tetramethylbenzidine (TMB) substrate solution (ThermoFischer, cat. # N301) in microplate and incubated at room temperature for 10 minutes with orbital shaking (700 rpm). Enzymatic reaction was stopped by adding 100 μL 2 M sulfuric acid and absorbance was measured at 450 nm.

### Spore germination

Spore germination measurements in the presence of L-alanine or mixture of asparagine, glucose, fructose and potassium cation (AGFK) were performed as follows. Spores were heat activated at 80°C for 10 min and diluted to an OD_600_ of 1 in 10 mM L-alanine and 10 mM Tris-HCl pH 7.5 (for L-alanine-induced spore germination) or in 10 mM Tris-HCl at pH 7.5 with 3.3 mM L-asparagine, 5.6 mM D-glucose, 5.6 mM D-fructose, and 10 mM KCl (for AGFK-induced spore germination). Germination was then monitored by following the loss of absorbance of spore suspensions at 600 nm.

### Production of recombinant M2eH-A-S-H in the bacterial expression system

Synthetic M2eH-A-S-H ORF was digested with *BamHI* and *EcoRI* enzymes and cloned into a commercial vector, pGEX2TK (GE Healthcare). The resulting plasmid, pM2eH-A-S-H-GST, was verified by restriction analysis and nucleotide sequencing. pM2eH-A-S-H-GST was used to transform BL21 *Escherichia coli* strain, and the recombinant strain was used to overproduce M2eH-A-S-H-GST after addition of IPTG (final concentration—1 mM). The protein was purified by affinity chromatography on glutathione resin (GE Healthcare). 0.2 mg of pure M2eH-A-S-H-GST protein was obtained from 0.25 L culture.

### Immunizations

Five groups of three mice (female, BALB/c, 8 weeks) were immunized orally with suspensions of recombinant spores expressing CotC-M2eH-A-S-H, CotB-M2eH-A-S-H, CotZ-M2eH-A-S-H, CgeA-M2eH-A-S-H fusions or control, non-recombinant spores (strain 168). A naïve, non-immunized control group was included. Oral immunizations contained 10^10^ spores in a volume of 0.2 mL and were administered by intra-gastric lavage on days 1, 3, 5, 22, 24, 26, 43, 45, 47. Serum samples were collected on day 61 from all animals in group.

### Detection of M2e-specific humoral response by western blot

15 μg of purified M2eH-A-S-H-GST was separated in polyacrylamide gel and further electrotransferred on nitrocellulose using iBlot^®^ 2 Dry Blotting System (Life Technologies). Sera obtained from each immunized animal were diluted (1:100) in TBS with 3% skimmed milk and served as a primary antibodies. A secondary polyclonal goat anti-mouse IgG-alkaline phosphatase (Sigma Aldrich, cat. # A3562) antibodies were used. Western blots were visualized developing with BCIP/NBT according to the manufacturer’s instructions (Thermo Scientific).

### Indirect ELISA for detection of antigen-specific serum

Plates were coated with 100 μl of the specific antigen per well (1.5 mg/mL in carbonate/bicarbonate buffer) and left at 4°C overnight. Antigen was M2eH-A-S-H-GST purified protein. After blocking with 0.5% BSA in PBS for 1 h at 37°C, serum samples were applied using a two-fold dilution series starting with a 1:20 dilution in ELISA diluent buffer (0.1 M Tris-HCl, pH 7.4; 3% (w/v) NaCl; 1.0% (w/v) BSA; 10% (v/v) sheep serum (Sigma); 0.1% (v/v) TritonX-100; 0.05% (v/v) Tween-20). Every plate carried replicate wells of a negative control (a 1:20 diluted pre-immune serum), and positive control (mouse monoclonal Influenza A m2 14C2; Santa Cruz Biotechnology, cat.# sc-32238). Plates were incubated for 2 h at 37°C before addition of goat anti-mouse IgG1, IgG2a or IgG2b (Sigma Aldrich, cat. # M5532, M5657, M5782 respectively). Plates were incubated for a further 1 h at 37°C followed by addition of rabbit anti-goat serum HRP conjugated (Sigma Aldrich, cat. # A5420). Plates were developed using the substrate OPD (o-phenylenediamine dihydrochloride; Sigma Aldrich). Reaction was stopped using 1 M citric acid and absorbance was measured at 450 nm.

### Indirect ELISA for detection of antigen-specific sIgA in lungs and trachea

The organs were collected and weighed before storage in the freezer at −20°C in a PBS solution (1 ml per g of tissue) containing 2 mM phenylmethylsulfonyl fluoride, 0.1 mg of trypsin inhibitor from soybean (Sigma Aldrich) per ml, and 0.05 M EDTA. Saponin (Sigma) was added to a final concentration of 2% (w/v) to permeabilize the cell membranes, and the samples were stored at 4°C overnight. The organs were spun down and the supernatant was analyzed for antibody content by ELISA as follows. Plates were coated with 100 μl of the M2eH-A-S-H-GST antigen per well (1.5 mg/mL in carbonate/bicarbonate buffer) and left at 4°C overnight. After blocking with 0.5% BSA in PBS for 1 h at room temperature, 20 μl of samples diluted in 80 μl ELISA diluent buffer (0.1 M Tris-HCl, pH 7.4; 3% (w/v) NaCl; 1.0% (w/v) BSA; 10% (v/v) sheep serum (Sigma); 0.1% (v/v) TritonX-100; 0.05% (v/v) Tween-20) were added. Plates were incubated for 2 h at room temperature before addition of goat anti-mouse IgA antibody, HRP conjugate (Cat.# 62–6720). Plates were incubated for a further 1 h at room temperature and developed using the substrate OPD (o-phenylenediamine dihydrochloride; Sigma Aldrich). Reaction was stopped using 1 M citric acid and absorbance was measured at 450 nm.

## Results and Discussion

### Construction and chromosomal integration of gene fusions

To obtain recombinant *B*. *subtilis* spores expressing influenza M2e antigen the synthetic M2eH-A-S-H sequence coding tandem repeat of 4 consensus sequence of human—avian—swine—human M2e was fused in frame with the spore coat protein genes *cotB*, *cotC*, *cgeA* and *cotZ*. The gene fusions retained the natural promoter of the appropriate coat protein gene to ensure proper timing of expression during the sporulation process. Gene fusions were integrated into the *B*. *subtilis* chromosome at the non-essential locus *amyE*. All gene fusions were integrated into the *B*. *subtilis* chromosome and individual clones for each transformation were tested by PCR, and named BTL20 (CotC-M2eH-A-S-H), BTL21 (CotB-M2eH-A-S-H), BTL23 (CotB-LINKER-M2eH-A-S-H), BTL22 (CotZ-M2eH-A-S-H), BTL24 (CotZ-LINKER-M2eH-A-S-H), BTL25 (CgeA-M2eH-A-S-H), BTL26 (CgeA-LINKER-M2eH-A-S-H) and used for further analysis.

**Fig 3:**
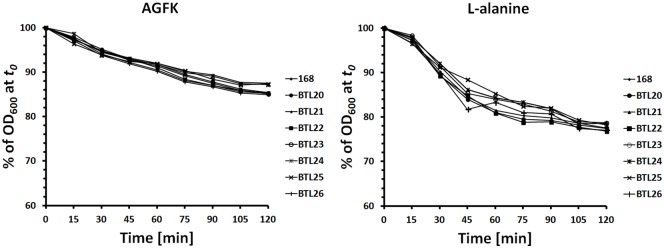
Germinantion of recombinant and parental strain induced by L-alanine and AGFK. Germination was followed by measuring the absorbance decrease at 600 nm of the spore suspension. Data are given as arithmetic mean from three independent experiments.

All of the recombinant strains and isogenic parental strain *B*. *subtilis* 168 showed comparable germination efficiencies (Fig 3). This results suggest that genetic recombination done in presented work did not affect the biology and properties of the spores produced by recombinant strains.

Constructed set of fusion proteins had to circumvent potential problems with their synthesis in the sporulating cells or placement in the structure of spore coat. We could not exclude the possibility that the constructed fusion proteins could gain toxic properties. Antigen attached to the coat protein could prevent the incorporation of a fusion protein into spore coat structure. Thus, we decided to screen four different spore coat proteins for their ability to serve as an anchor motif for influenza virus antigen exposition on *Bacillus subtilis* endospores. We used as well 11-aa linker forming helical structure to overcome potential problems mentioned above.

### Spore coat expression and surface display

**Fig 4:**
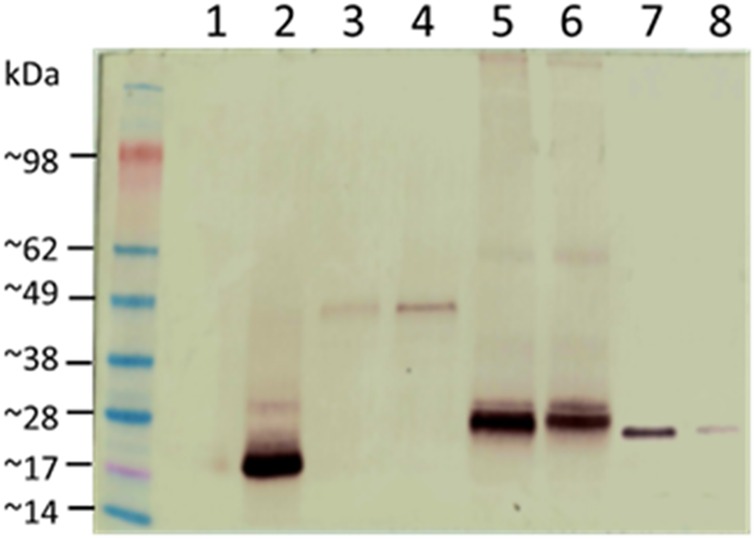
Western blot analysis of fusion genes expression. Spore coat proteins were extracted and analyzed by western blotting with anti-influenza M2 mAb. Numbers of lanes refer to the strain used in experiment: 1-*B*. *subtilis* 168; 2-BTL20(CotC-M2eH-A-S-H, ~20 kDa); 3-BTL21(CotB-M2eH-A-S-H, ~43 kDa); 4-BTL23(CotB-LINKER-M2eH-A-S-H, ~44 kDa); 5-BTL22(CotZ-M2eH-A-S-H, ~28 kDa); 6-BTL24(CotZ-LINKER-M2eH-A-S-H, ~29 kDa); 7-BTL25(CgeA-M2eH-A-S-H, ~26 kDa); 8-BTL26(CgeA-LINKER-M2eH-A-S-H, ~27 kDa).

**Fig 5:**
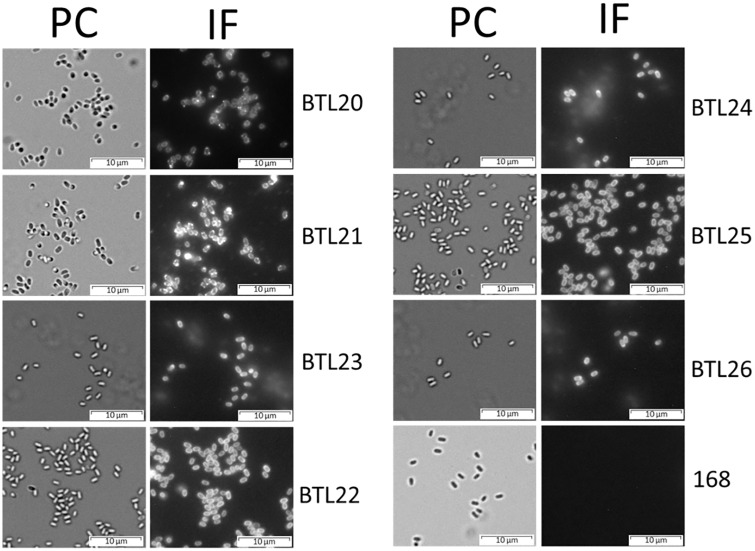
Immunofluorescence staining of recombinant spores. Purified spores were incubated with anti-influenza M2 mAb, followed by anti-mouse Cy3 conjugates. Spores were visualized by phase contrast (PC) and immunofluorescence (IF) microscopy. The same exposure time was used for all IF images.

The localization of fusion proteins in the spore coat was tested by western blotting with mouse monoclonal (14C2) anti-influenza A M2 antibodies (Santa Cruz Biotechnology). In case of all tested recombinant spores we could observe a specific signal. The recombinant proteins observed showed apparent molecular weights that correlated with the deduced molecular weights (Fig 4). The surface localization of fusion proteins was analyzed by immunofluorescence microscopy of dormant spores of wild type and recombinant strains using anti-influenza A M2 (Santa Cruz Biotechnology) primary antibodies and anti-mouse IgG-Cy3 (Jackson Immuno Research) as secondary antibodies. We observed a fluorescent signal from purified dormant spores of all tested recombinant spores (Fig 5).

These results show that all of the spore coat proteins used in our study are able to display viral antigen on the spore surface, however with different efficiency for each coat protein.

### Antigen expression efficiency and accessibility

**Fig 6:**
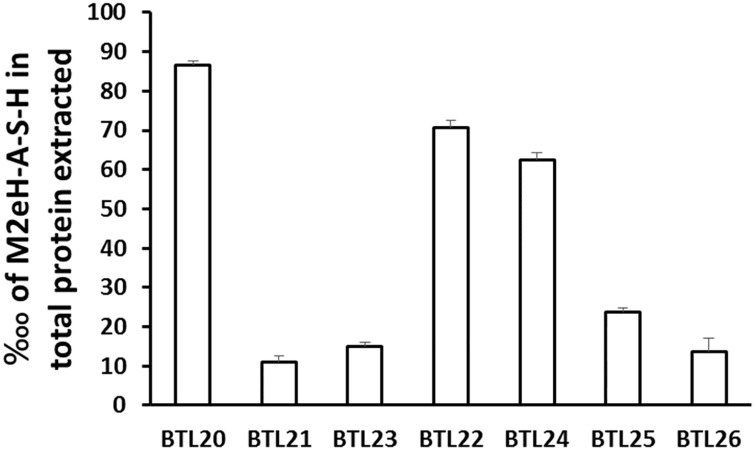
Efficiency of antigen expression in constructed strains. Data are given as arithmetic mean with ±SD from three independent experiments.

A quantitative determination of the amount of M2eH-A-S-H present on the surface of *B*. *subtilis* spores was obtained by dot-blot experiments using serial dilutions of purified M2eH-A-SH and coat proteins extracted from spores of the recombinant strains. The proteins were subjected to anti-M2e antibodies, followed by alkaline phosphatase-conjugated secondary antibodies and the blots were developed using BCIP/NBT (Thermo Scientific). Densitometric analysis indicated that the CotC—M2eH-A-S-H fusion protein amounted to 0.86 ± 1.52%, CotB—M2eH-A-S-H to 0.11 ± 1.00%, CotB—linker-M2eH-A-S-H to 0.15 ± 2.00%, CotZ—M2eH-A-S-H to 0.70 ± 2.08%, CotZ-linker-M2eH-A-S-H to 0.62 ± 2.51%, CgeA—M2eH-A-S-H to 0.23 ± 3.51% and CgeA-linker-M2eH-A-S-H to 0.13 ± 2.30% of total coat proteins extracted (Table 3). Our observations show that different spore coat proteins deliver an antigen to the spore coat with different efficiency with CotC and CotZ being most efficient. We could not observe that applying a linker improved spore coat expression of a fusion protein distinctly (Fig 6). According to the data obtained by Hinc and Negri [34,35] we can conclude that efficiency of antigen loading into spore coat depends most probably on both the passenger protein and the properties of the obtained fusion, and does not necessarily simply refer to the anchoring properties of coat protein. This observation indicate that generation of recombinant spores presenting antigens should include preparation of panel of fusion proteins to select most efficient anchoring coat protein.

**Fig 7:**
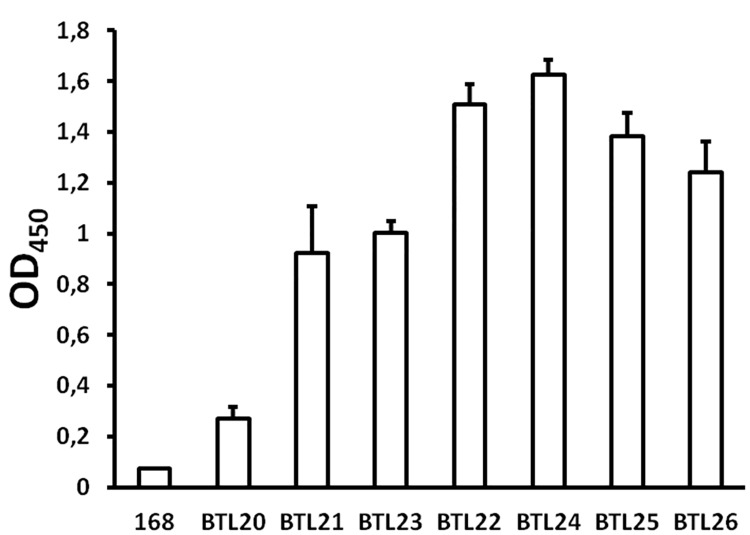
Antigen accessibility to antibodies in different constructs. Intact spores were incubated with mouse anti-M2e primary antibody followed by incubation with goat anti-mouse IgG peroxidase secondary antibodies. 10^4^ spores were incubated in TMB substrate solution to develop color reaction. Absorbance was measured at 450 nm and data are given as arithmetic mean with ±SD from three independent experiments.

Spore coat is a complex, multi-layer structure thus different anchoring proteins can provide variable accessibility of the presented antigen to the immune cells or antibodies. To test this, we performed ELISA with intact spores. The results show various degrees of displayed antigen accessibility to the antibodies depending on the anchoring protein (Fig 7). The highest level of antigen accessibility feature spores BTL22/24 and BTL25/26 presenting M2eH-A-S-H in fusion with CotZ/CotZ-LINKER and CgeA/CgeA-LINKER coat proteins respectively. Spores produced by strains BTL21/23 harboring M2eH-A-S-H in fusion with CotB/CotB-LINKER showed diminished accessibility of presented antigen compared with strains BTL22/24/25/26. Dramatic decrease in antigen accessibility could be observed in case of spores BTL20 (CotC-M2eH-A-S-H) which showed 4 times lower OD_450_ value compared with strains BTL22/24. When comparing antigen expression levels with providing accessibility of the presented antigen by different spore coat proteins, obtained results appear to be inconsistent to some extent. Protein CotC features the highest antigen load into spore coat although provides the lowest accessibility of presented antigen compared with other anchoring proteins. In contrary, CgeA provides high antigen accessibility however its antigen loading properties are diminished compared with other anchoring proteins. This observation is most probably due to the different locations which are naturally occupied by anchoring proteins in the spore coat structure. Based on the literature spore coat proteins CotZ and CgeA are formers of the outermost layer of the coat called crust [36]. This could explain high accessibility of the antigen to the antibodies despite its low abundance in the spore coat in case of the BTL25/26 spores. CotB and CotC proteins are found deeper in the spore coat structure called outer coat thus accessibility of presented antigens provided by those proteins can be diminished compared with CgeA and CotZ. Moreover, it was shown that the CotC protein features uneven distribution on the surface of spores being more abundant at the F-pole of the spore [37].

### Immune response to recombinant spores

**Fig 8:**
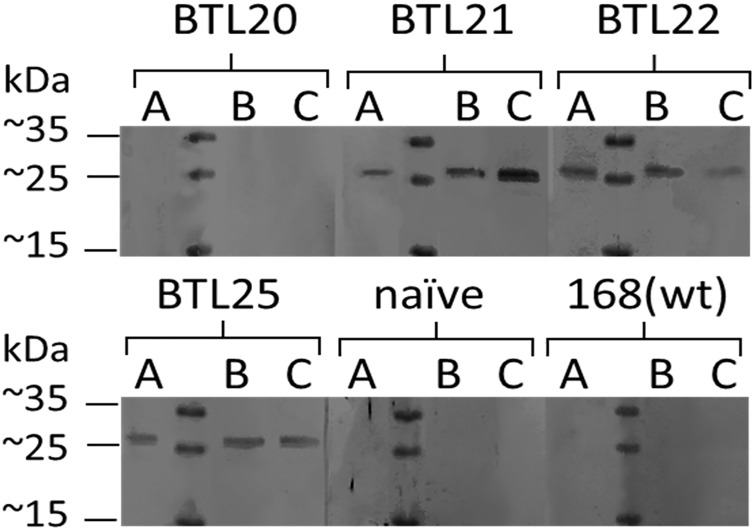
Western blot analysis of sera from immunized mice. 5 groups of 3 mice (A, B, C) were orally immunized with recombinant spores of strains *B*. *subtilis* 168; 2-BTL20(CotC-M2eH-A-S-H), BTL21(CotB-M2eH-A-S-H), BTL22(CotZ-M2eH-A-S-H) or BTL25(CgeA-M2eH-A-S-H). A 15 μg of recombinant M2eH-A-S-H-GST was separated in polyacrylamide gel and further electrotransferred on nitrocellulose which was further incubated with sera from immunized mice as a primary antibodies. Blots were developed using anti-mouse alkaline phosphatase conjugates. Figure is a merge of 20 different single-lane blot strips.

Recombinant spores produced by strains BTL20 (CotC-M2eH-A-S-H), BTL21 (CotB-M2eH-A-S-H), BTL22 (CotZ-M2eH-A-S-H), BTL25 (CgeA-M2eH-A-S-H), were selected to test their immunogenic properties. A water suspensions of selected recombinant spores were orally administered to mice. On day 61 of the immunization blood samples from all animals of each groups were collected to obtain sera. The specific antibodies production by mice immunized with recombinant spores was tested using western blot analysis. A specific humoral antigenic response was detected in all animals immunized with spores harboring CotB/CotZ/CgeA-M2eH-A-S-H fusion protein (Fig 8). We did not observe any specific signal in case of sera obtained from animals immunized with spores harboring CotC-M2eH-A-S-H fusion protein. Sera obtained from the naïve group and control group gave negative results. Surprisingly, spores harboring fusion CotC-M2eH-A-S-H and showing the highest antigen load failed to induce M2e specific antibody production in mice. Although the mechanism of developing immune response elicited by recombinant endospores is not clear, we propose following hypothetical explanation of the observed phenomenon. To stimulate the immune system with an antigen delivered orally on spores, presented antigen needs to get in direct contact with immune cells. It can happen in two ways: (i) Lack of antibody production induced by spores harboring CotC-M2eH-A-S-H could be a result of problematic accessibility of the antigen to the B cells receptors. In our study we showed that CotC provides the lowest accessibility of M2e antigen for antibodies in *in vitro* experiments. (ii) Spores are phagocytosed within the gastrointestinal tract by cells of the gut-associated lymphoid tissue. Inside the phagocytic cells spores are processed in a manner which allows for an antigen presentation by antigen-presenting cells. Spores germinate within intestine what results in a spore coat shedding. Spore coat debris is taken up by immune cells and further processed for an antigen presentation. In both cases spore coat needs to be digested for extraction of carried antigen. Spore coat is known to be a feature which facilitates resistance against many unfavorable and destructive agents, such as gastric fluid. Thus, the spore coat digestion by the immune cells could be challenging.

**Fig 9:**
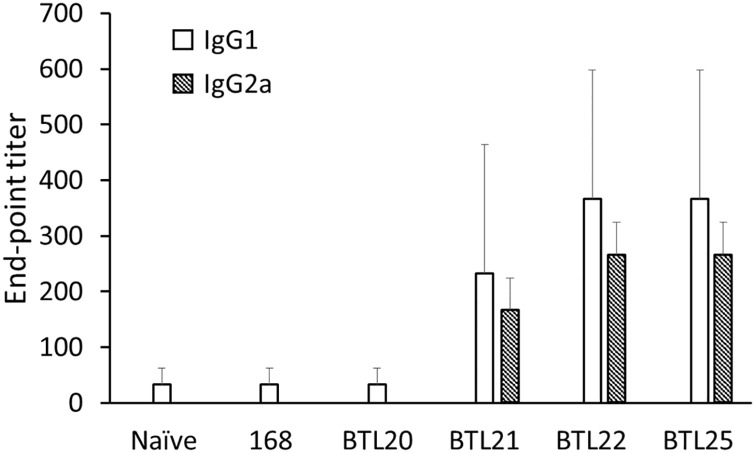
ELISA of anti-M2e IgG isotyping. IgG1, IgG2a and IgG2b subtypes were tested using mouse sera from 61^st^ day of immunization. The titers were expressed as the endpoint dilutions that remained positively detectable. The data are presented as arithmetic mean ±SD of 3 mice per group.

To further investigate the antibody production in immunized mice we conducted ELISA antibody titration with isotyping of IgG subclasses. The antibody titer was established in the range of 1:100 to 1:500 and only for IgG1 and IgG2a subclasses (Fig 9). Established antibody titers suggest the low immunogenicity of tested recombinant spores. Observed response probably would not provide efficient protection against viral infection. Nevertheless, our study shows that spores harboring low immunogenic M2e antigen can induce specific antibody production without use of adjuvant. In contrast, use of *B*. *pertussis* as a live M2e antigen carrier failed to induce antibody production in intranasally immunized mice [38].

Influenza virus infection begins in respiratory tract thus production of secretory antibodies in this part of the body is important to prevent the viral infection. We examined lungs and trachea of immunized mice in terms of specific sIgA production however we did not observe any immune response (data not shown). The mucosa-associated lymphoid tissue represents a highly compartmentalized immunological system. This compartmentalization results in developing specific, local immune response with the highest IgA production at the site of antigen administration [39]. In our study we used the oral immunization what could be inefficient in inducing immune response in mucosa of respiratory tract. Thus an intranasal immunization should be considered for induction of lgA production in respiratory tract.

## Conclusion

The development of an efficient vaccine against influenza virus infections seems to be a real challenge. In spite of extensive studies focused on generation of universal influenza vaccine, satisfactory solution still has not been found. In our work we show that *B*. *subtilis* spores can serve as a platform for influenza virus antigen exposition. All spore coat proteins used in this study to construct recombinant spores managed to anchor the M2e influenza virus antigen on the spore coat surface. Immunogenicity of the recombinant spores was tested by oral administration in mice what gave positive results. We could isolate specific antibodies from sera of immunized mice. However established antibody titers were relatively low suggesting that developed immune response wouldn’t be protective. Nevertheless, in our opinion, the present system remains attractive and should be improved in order to increase its immunogenicity.
